# Accompaniment in the healthcare sector: a systematic review and concept analysis

**DOI:** 10.3389/fmed.2026.1724133

**Published:** 2026-02-20

**Authors:** Cristina Antón-Rodríguez, Maleny Medina, Pilar Rodríguez, Ángel Barahona, Santiago Álvarez-Montero

**Affiliations:** 1 School of Medicine, Universidad Francisco de Vitoria, Madrid, Spain; 2 Institute of Accompaniment, Universidad Francisco de Vitoria, Madrid, Spain; 3 Department of Humanities, Universidad Francisco de Vitoria, Madrid, Spain

**Keywords:** accompaniment, concept analysis, healthcare humanization, professional relationship, systematic review

## Abstract

**Background:**

Accompaniment is increasingly recognized across professional practices as a key relational process, yet its meaning varies widely. Clarifying this concept is essential for theoretical development and practical application in healthcare and related disciplines. This study aimed to analyze the concept of “accompaniment,” distinguishing its everyday use from its scientific meaning, and to establish an operational definition with defining attributes.

**Methods:**

A concept analysis was conducted following the eight-step model of Walker and Avant. Everyday uses were identified through dictionary searches and online engines (Google, ChatGPT-4, and Copilot). Professional uses were determined through a systematic literature review performed in accordance with the PRISMA 2020 Statement. Eligible publications presented original definitions or descriptions of accompaniment and were written in English, French, Italian, Spanish, or German. Searches were conducted in PubMed, Embase, and Cochrane Central. Methodological quality was assessed using the JBI Critical Appraisal Tool for textual evidence. Attributes were extracted through a triangulated interpretative inductive analysis to derive a concise definition. Model, related, contrary, and borderline cases, as well as empirical referents, were developed.

**Results:**

From 378 records, 19 publications (16 main authors) met the inclusion criteria. The most frequent attributes described as accompaniment is a personal and ethical relationship oriented toward human development through a shared process that fosters a humanized social climate. The resulting definition was Accompaniment is a relational process between two or more people who share a path toward reciprocal development, in which at least one intentionally offers appreciation, presence, acceptance, and support to promote the other’s full growth while respecting dignity, freedom, and context.

**Conclusion:**

Accompaniment differs from mentoring, coaching, counseling, motivational interviewing, preceptorship, and shared decision-making. It is a transversal, interdisciplinary concept that can contribute to humanizing healthcare and supports the development of future measurement tools.

## 1 Introduction

Quality human relations are essential. They are associated with a longer, healthier, and happier life. Belonging to a group can help regulate stress. Conversely, an undesired solitary life and isolation are stressful situations (4). Human beings are born, grow up and develop within and thanks to a relational framework (5). Experiencing encounters with one another is key, as they result in dynamisms that have a creative and enriching potential for all (6). There is widespread evidence that professionals who have experienced mentoring relationships that fostered their professional development identify mentoring as a key factor in their working life, whereas those without such experiences tend to perceive it less positively (7). High-quality relationships are also associated with a lower risk of burnout, particularly in the dimension of personal development (8).

Across multiple disciplines (medicine, nursing, psychology, education and social work), accompaniment is increasingly recognized as a relevant component of professional practice. Since the 1970s, a steady rise in the use of the term “accompaniment” has been observed in PubMed-indexed publications, with the number of articles published in the last 5 years equaling those of the preceding eleven. This growth in academic interest coincides with the COVID-19 (SARS-CoV-2) pandemic, which has had a profound impact on mental health and wellbeing worldwide (9). Public health measures such as lockdowns severely restricted social contact (10), highlighting both the central role of human relationships in healthcare and the detrimental effects of loneliness on physical and emotional wellbeing (11).

The word “accompaniment” derives from the Latin compound *cum panis* (cum, “with”; panis, “bread”), conveying both the idea of walking together and of sharing bread. It evokes the act of sharing something essential for sustaining life, while metaphorically moving in the same direction (12).

In the Judeo-Christian tradition, accompaniment appears from the outset as a fundamental dimension of human experience, associated with a people journeying together (as YHWH with Israel in the wilderness) and with companions on the road, such as the disciples on the way to Emmaus described in the Gospel of Luke (13). During the Middle Ages, it became a common practice in monastic and spiritual life. In the sixteenth century, Ignatius of Loyola systematized accompaniment through the Spiritual Exercises, establishing it as a pedagogical tool to support personal discernment and vocation (14). In more recent times, the term gained renewed prominence in the 1970s through Archbishop Óscar Romero and has been consistently emphasized by Pope Francis as a central element of pastoral practice (13).

In the history of medicine, accompaniment has rarely appeared as an explicit element of healing practice, although it has implicitly been present. In ancient India, the *Caraka Samhita* urged physicians not to abandon the patient; Confucianism emphasized the virtue of *ren* (love for humanity), and Buddhism highlighted compassion for the suffering (15). Similarly, the Hippocratic tradition, centered on *tékhne iatriké*, conceived medical practice as a form of help marked by a “maternal disposition” aimed at making illness more bearable (16, 17). In the Middle Ages, this philanthropic orientation evolved into *caritas*, grounded in the belief that “in the sick person there is Christ” (18). In the eighteenth century, John Gregory stressed that physicians should show affability and gentleness, “suffering with” the patient and not abandoning them, even at the end of life (19).

Laín Entralgo later explicitly introduced the notion of accompaniment into the doctor–patient relationship, describing it as a bond of companionship in which one accompanies and the other is accompanied, summarized in the maxim: “to cure frequently; to relieve always; to comfort often; and to accompany at all times” (20).

From the 1990s onwards, accompaniment practices became more visible not only in healthcare but also in social work, education, the judicial system and organizational contexts (21). In a meaning close to its etymological roots, Behforouz defined medical accompaniment as “walking alongside” patients, particularly vulnerable groups, through sustained support, trust-building and collaborative action to overcome structural barriers to health (22).

At the Universidad Francisco de Vitoria, where this study was conducted, personal accompaniment constitutes a core element of the institutional mission and educational model, understood as advancing together in learning, research, management and personal development through relationships of encounter (23). This perspective is grounded in a personalist anthropology that conceives the human person as a being-in-relationship, who grows through encounters with reality, self, others and transcendence (24).

Such an approach to human relations may positively influence job satisfaction, professionalism, career development and protection against burnout, and may contribute to the humanization of healthcare in clinical, educational, research and managerial settings. From a theoretical standpoint, accompaniment can be situated within interpersonal and communicative systems, in line with King’s theory of systems, concepts and processes (25). However, validated instruments to assess this construct are currently lacking.

Moreover, despite its increasing relevance, accompaniment is understood with varying meanings across professional fields and is often conflated with related concepts such as tutoring, supervision, coaching, counseling, mentoring, motivational interviewing or shared decision-making. A conceptual analysis is therefore required to clarify its essential and peripheral components. Following Walker and Avant (26), conceptual analysis is understood as a systematic process aimed at identifying the structure and function of a concept, allowing for a precise operational definition.

Accordingly, the aim of this study is to analyze the concept of accompaniment in order to distinguish its everyday and scientific uses and to identify its defining attributes through a systematic review of the literature, with a view to developing measurement tools applicable to the health sciences. We hypothesize that accompaniment constitutes a distinct construct, not fully reducible to related professional relationships such as mentoring, tutoring or counseling, and that its clarification will facilitate scientific communication and future empirical assessment.

## 2 Methodology

To carry out the analysis of the concept of accompaniment, the eight-step model proposed by Walker and Avant (26) was used. This method begins with the selection of the concept of interest - in this case, accompaniment -as outlined in the Introduction and Justification section. This is followed by identifying the different uses of the concept through dictionary definitions, everyday language and its technical application in various disciplines, by means of a systematic review of the literature.

From this analysis, the defining attributes of the concept are determined, i.e., those essential characteristics without which the concept would lose its identity. Based on these attributes, a representative model case of the concept in the scientific field may then be devised. The process continues with the elaboration of additional cases that allow for a more precise delimitation of the concept. Finally, the identification of antecedents and consequences is carried out, i.e., the factors that precede its occurrence and the effects that derive from its manifestation (26).

Three procedures were used to identify the uses of the concept:

A first search in dictionaries, consisted in looking up the term in the Diccionario de la Lengua Española (27) the Cambridge Dictionary (28) and the Dictionnaire de l’Académie française (29).

A second search for everyday uses was carried out using the Google search engine (1) (https://www.google.com), Chat GPT 4th (2) and Copilot(3) with the prompt *I am *a researcher at a Faculty of Medicine in Madrid and I am working on the analysis of the concept of “Accompaniment”; I need you to indicate the most common uses of accompaniment in any social and everyday environment in Europe*. These tools were not employed as primary data sources, but as supplementary instruments to support the initial mapping of meanings, in line with their growing use in qualitative research for horizon scanning and hypothesis generation.

To address the well-known limitations of generative AI—such as lack of source traceability, potential hallucinations, cultural bias and dependence on training data—the information obtained was not accepted at face value. Instead, AI-generated outputs were: (1) contrasted with definitions from authoritative dictionaries in three languages, (2) triangulated with results from the systematic literature review, and (3) critically discussed by the research team to assess conceptual coherence with the theoretical framework of Walker and Avant’s concept analysis. Only uses that were consistent across these three sources were retained.

A third search consisted of a systematic literature review (30) following the PRISMA 2020 Statement (Preferred Reporting Items for Systematic Reviews and Meta-analysis) (31). The studies and scientific papers had to include an original definition of accompaniment describing the attributes of the concept published in scientific journals or books in English, French, Italian, Spanish, or German. The PubMed, Embase, and Cochrane Central databases were consulted using the search strategies detailed in the Supplementary materials. In addition, a manual search of studies by relevant authors in the field of accompaniment was carried out, consulting with experts and scholars on the subject (people of different nationalities and scientific areas who had taken part in the I and II International Conference on Accompaniment held at the UFV: The educational power of accompaniment and accompaniment in the world of health: the healing impact of relationships). The last search session was dated February 20th, 2025. The methodological quality of the included textual sources (e.g., book chapters, expert opinions and theoretical essays) was assessed using the JBI critical appraisal tool for textual evidence of experts’ opinions. This tool examines six criteria: clear identification of the source, authority of the author, centrality of interest to the target population, logical argumentation, reference to existing literature and justification of discrepancies with other sources.

Once the records of the searches were obtained, a systematic review was created in rayyan.ai^1^ from which duplicates were removed and the selection of publications meeting the inclusion criteria was carried out in two phases by two independent authors (SAM and CAR). The first screening was by title and abstract. From those selected as potentially eligible, the full texts were obtained. By the subsequent analysis of the information therein, their inclusion or otherwise was decided upon. When included, the accompanying definitions were extracted to aid in the search for the concept’s defining attributes.

Following Walker and Avant’s methodology, the attributes of the concept most frequently mentioned were selected, thereby forming the core of the conceptual analysis (26). For this purpose, a triangulated interpretative inductive conceptual analysis was carried out by two of the authors (SAM and CAR) based on literal texts of the definitions of accompaniment provided by the systematic review. This analysis was oriented toward the generation of categories, the aim being that from them the attributes of the concept of accompaniment may subsequently be extracted (32). The attributes act as criteria for recognizing the emergence of this concept and contribute to understanding its core meaning. The smallest number of attributes was identified in order to correctly differentiate the concept of accompaniment from other surrounding concepts.

Subsequently, a model case which included all the identified defining attributes was described, thereby developing a meaning of the concept as a whole in a clear and contextualized manner. In addition, further cases were presented, such as cases similar to, related or contrary to the concept. These are intended to help understand the internal structure of the concept, clarify its boundaries and show its use in various contexts (26, 32).

The antecedents and consequences of the concept were then identified and described. Antecedents are the events or incidents that must occur before the concept is produced, while consequences are effects or events that occur as a result of its manifestation. It is important to note that neither the antecedents nor the consequences should contain the defining attributes (26).

Finally, the empirical referents of the concept were defined, understood as observable indicators that make it possible to recognize the presence of the defining attributes in real situations. These empirical referents are essential to translate the concept into measurable terms, facilitating its application in assessment, intervention and instrument development contexts (33). This is because they are clearly linked to the theoretical basis of the concept, thus contributing to both the content and construct validity of future measurement tools (34).

## 3 Results

### 3.1 Uses of concept

In the Spanish, English, and French dictionaries reviewed, the term accompaniment has different meanings. They share the meaning of the action of accompanying, i.e., the idea of “to be or go in the company of another person or persons” or “to join or add something to something else.” It often refers to people, things (especially of a culinary and musical nature) or even events. In Spanish the meaning is extended to someone’s qualities (“le acompaña la fortuna,” he is fortunate), to share someone else’s feelings (“te acompaño en el sentimiento,” *my condolences*), or to the very people who accompany someone (“vino con mucho acompañamiento,” *he arrived with many people*) (35). In English, it is predominantly used as a musical accompaniment and in events that happen simultaneously (36), while in French it is used as “to escort someone out of deference, to guide, protect or watch over them” (29).

**FIGURE 1:**
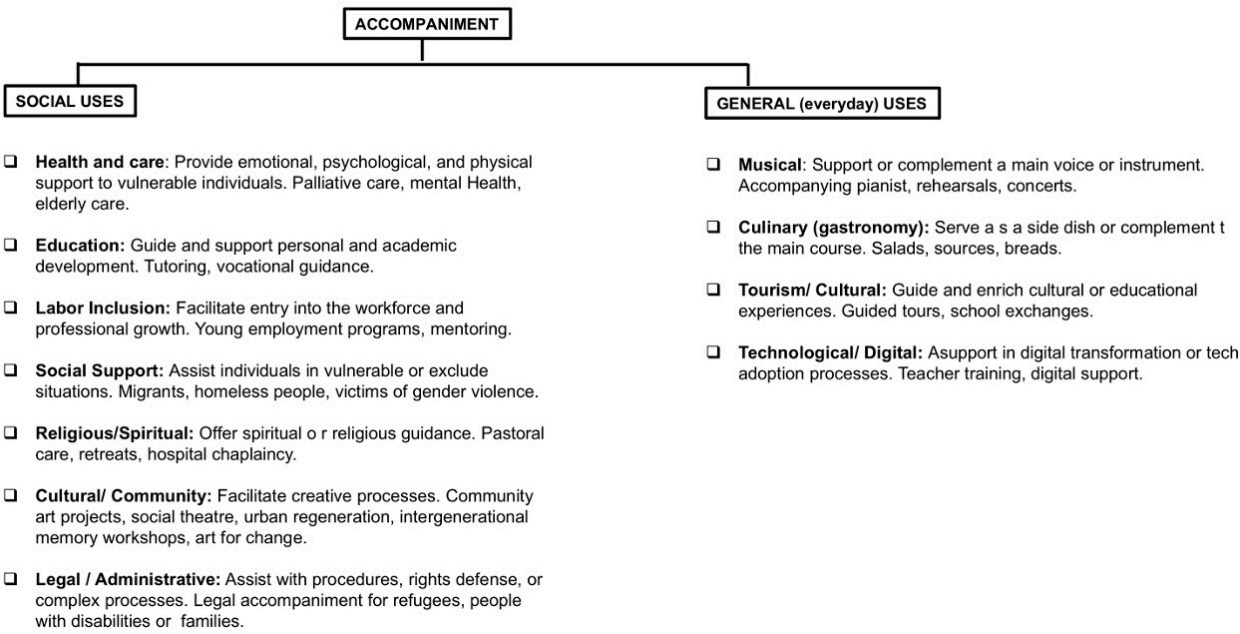
Conceptual diagram: social and general uses of the concept of “Accompaniment” in Europe. Own elaboration based on the analysis of European sources, with support from ChatGPT (Open AI, 2025).

Figure 1 shows the schematic with the description of the purpose and examples of social and everyday uses obtained by consulting the search engine (Google, s. f.), powered by Chatgpt (2) and Copilot (3).

**FIGURE 2:**
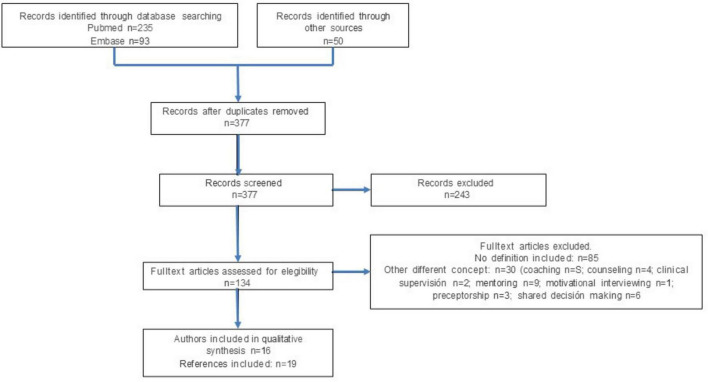
Flow diagram of the search conducted in PubMed and Emabase.

Figure 2 shows the flow chart of the systematic review carried out in the Pubmed and Embase databases, where a total of 235 records were obtained from the former and 93 records from the latter. To these were added another 50 records obtained through manual searches, recommended by experts in accompaniment (in this we include both scientific articles and books or specific chapters therein).

These records were uploaded to Rayyan’s tool (see text footnote 1) and the 19 duplicates detected were removed in a mixed (automatic and manual) manner.

In the first title and abstract screening of the 377 records identified, 243 records were excluded because they failed to meet the inclusion criteria. The full texts of the 134 articles or books that did meet the criteria were obtained. In this full-text eligibility phase, 85 were excluded because they did not include a definition of accompaniment or the definition referred to in the text was not original to the authors.

Thirty articles dealing with concepts only related to accompaniment were also excluded: 5 on coaching, 4 on counseling, 2 on clinical supervision, 9 on mentoring, 1 on motivational interview, 3 on preceptorship, 6 on shared decision making. References and grounds for exclusion are detailed in see Supplementary materials “Excluded by fulltext_reference and reason.” Finally, 19 publications remained, corresponding to the works of 16 main authors (12, 13, 21, 37–51). See Supplementary materials characteristics and quality of included studies.

Overall, the quality appraisal showed that most of the included sources fulfilled the majority of the JBI criteria. All documents clearly identified their authors and demonstrated logical coherence and grounding in existing literature (Items 1, 4, and 5). The authority of the authors in their respective fields was generally well established (Item 2). The criterion least consistently met was the explicit justification of discrepancies with alternative perspectives (Item 6), which was rated as unclear in a small number of sources. These results indicate that the conceptual material included in the analysis is supported by expert authorship and coherent argumentation, although explicit engagement with competing viewpoints was not always reported.

The systematic review showed that the term “accompaniment” has been used in many different professional fields, such as psychiatry (52), psychosocial support to vulnerable groups (53), leadership (54), business entrepreneurship (55), and in the development of educational models (56).

One of the first original definitions was proposed in 2004 by Maela, with one of the broadest and deepest linguistic studies, synthesizing her work with the notion of accompaniment as joining up with another and going where they are going together (21). In 2012, Farmer, from a development aid context, states that to accompany someone is to go with him or her, to be present on a journey with a beginning and an end for a period of time (41). Another early definition was put forward in 2016 by Simard, who stated that accompaniment is a cross-cutting practice of relating to someone (not something) in which there is presence, bonding, trust and sharing of a common humanity, with the aim of supporting the other toward a direction for which he or she needs to go (43).

One of the most comprehensive and elaborated definitions - this time with a marked philosophical language - is that of González Iglesias: a harmonized path of encounters of freedoms (and of disagreements and moments of conflict that avoid the occurrence of a merely superficial harmony) with oneself, with others, with reality and with the transcendent, throughout a significant and continuous time, oriented towards the fullness of the person, which reaches and illuminates our human nature, our way of being human and our way of life (49).

Several specific definitions have also been proposed for pedagogy: in the field of health education, the *South African Nursing Council* defined accompaniment as the help and support provided to students by the professional nurse or midwife in order to develop competent and independent professionals (37). It has also been defined as a valuable strategy that effectively strengthens teaching performance by seeking to create an atmosphere of harmony and affective communication in the classroom between the person who accompanies and the person who is accompanied, in order to obtain more effective learning (46). Another definition understands accompaniment as the relationship offered by the educator to the learner as a means of assistance, generating personal growth in both (49); another understands the concept as a wanting and knowing how to walk along the path of the learner’s development - and therefore also in his or her professional and personal development (48).

In palliative care, accompaniment has been understood as the result of being present in the in-depth spiritual work carried out by therapists (45). To accompany means to be with another person, evoking an act of sharing something with a person, metaphorically going in the same direction as them. From this perspective, to accompany at the end of their life means to participate in a shared experience with the intention of being present and entering into encounter (50).

From a Christian pastoral point of view it has been defined as a way of forming and deepening community and discipleship, reflecting a deep trust in the guiding presence of the Holy Spirit in friendships and communities open to the influence of grace, being a form of long-term fellowship that is fostered by physical proximity and a willingness to engage in open and honest dialogue in a spirit of mutual respect (13).

### 3.2 Defining attributes

Attributes are concept properties that are most frequently associated with it (see Table 1). They provide a more comprehensive understanding of it and help to differentiate the concept of accompaniment from those that are similar in meaning.

**TABLE 1:** Attributes that authors include in their definitions.

References	Personal relationship	Oriented toward personal growth	On a path or process	Ethical	Humanizing
(37)**, as cited in** (40)	1	1	0	1	0
(21)	1	1	1	1	1
(38)	1	1	1	1	0
(39)	1	1	1	1	0
(46)	1	1	1	0	1
(41)	1	0	1	1	0
(42)	1	1	0	1	1
(43)	1	1	1	1	1
(12)	1	0	1	0	1
(13)	1	1	1	1	1
(45)	1	1	1	1	1
(47)	1	0	0	1	0
(48)	1	1	1	1	1
(49)	1	1	1	1	1
(50)	1	0	1	1	1
(51)	1	1	0	1	1
Total	16	12	12	15	11

The attributes that have proved most useful in identifying specific accompaniment attributes that are different from other types of relationship have been the following:

#### 3.2.1 Accompaniment is a personal relationship

Accompaniment is a type of relationship (43), i.e., a situation in which there is interaction between people. It is “going with” another in an “intentional” and “non-directive” way, unlike other types of practices (21), a being with and for another (12). It has also been expressed as “inhabiting” the spaces that the other “inhabits” (41).

Accompaniment is understood as a specifically a personal relationship, i.e., a relationship with “someone” not with “something,” which implies that it is “essentially egalitarian,” (43) horizontal and non-hierarchical in nature (21). It is a relationship that does not seek to dominate or remedy, but to be present and available for what the other needs to discover or do for themselves (21). Being of a personal nature, it is a unique and unrepeatable “I-you” (49), as each person is unique and unrepeatable and the main focus is on the person, not the problem itself. It is carried out by sharing stories, voicing grievances and expressing aspirations (13).

#### 3.2.2 It is oriented toward personal development or growth

The accompanied and the accompanying are going in the same direction (50), as the accompanied person is moving in a certain direction and needs support to reach their destination (43). Accompaniment is oriented toward responding to the human condition of openness, to the possibility of continuous development or growth (49, 51) - something that has also been put forward as the search for a “vocational fulfillment” (49). Personal growth is something integrally open to all human faculties and dimensions, including the spiritual or transcendent dimension (42, 45), which manifests itself as a constant search for the meaning of one’s life (49). It involves working on an emotional level, that of motivation, on a cognitive level and on a social level to address the individual as a whole (38, 49).

This can take the form of any facet of personal life, including those related to work (39), personal health (42), education (46) or in general of a professional nature (48). Hence, accompaniment can be a cross-cutting practice across all professions, disciplines and people’s life contexts (43).

#### 3.2.3 It is an ethical relationship

It is an ethical relationship (21, 43, 51) in a context of selfless interest, of gratuitousness (49), in which the accompanying person assumes a position of moral responsibility towards the person being accompanied (43).

The companion is able to experience a genuine interest in the other person (45), an interest that is not arise out of mere curiosity, but is based on recognition and respect (38) for the dignity of the other, as well as care and concern for what the other person may experience (43).

The accompanying person is in a position of caring for the other (43, 47), which goes beyond the superficiality of behavior, as it includes the realm of intentions (50). It shows a non-violent attitude, because it preserves human dignity from any violation and maintains a posture of openness and acceptance of fragility (43).

The companion offers his or her company (47), which translates into an offer to help (50), an availability (49) and intentional and continued presence (12, 13, 41, 50). It is a compassionate presence, which requires a contemplative gaze (45), open to dialogue (13) and to the totality of the other person (49).

Non-violence begins with deep listening to the whole patient, contemplating mind, body and spirit (45); listening is empathic (43), relying on silence in communication (45) and helps the person being accompanied to ask questions (12, 49).

Whoever accompanies acts with respect for personal freedom (21, 49), letting others be themselves, finding the right distance to be taken, knowing when and how to step back, to reserve oneself (38). It provides the map, references and tools for the other to express the direction he or she chooses (21). Moreover, it adapts to the pace and process of the person (43). For all these reasons, it greatly differs from authoritarian or manipulative forms of support (38).

The companion says “I will go with you and support you on your journey, wherever it takes you; I will share your destiny for a while” (41). This has more to do with art - with an interpretation of what the other is experiencing and presence - than with technique (50). It does not rely so much on mastery, but much more on confidence (43) - although it does require both.

Accompaniment is not to be seen as primarily a problem-solving relationship, (49) but rather as a support for people in the process of overcoming their problems (43).

#### 3.2.4 It is a path or process

The relationship, oriented towards mutual growth, metaphorically takes place on a path (43) or journey (50). This requires concrete spaces (21, 48) in which encounters with others, with oneself, with reality and with the transcendent, can take place (49). At times the road is a process in which disagreements arise that, at their best, avoid resorting to a merely superficial harmony (49).

All of this takes place within a unifying framework of linking and transition, monitoring and sharing (21), a dynamic of collaboration and sharing (43). On this path, experiences are felt together (43, 48) for a significant duration of time (13, 38, 49). The process has a direction along which the unpredictable is accepted; it is a model of change in which there is an a support that helps and empowers the person accompanied (38). That said, it is also a time when change may not necessarily occur (21).

On the path or process there is experience of reciprocal development as, although there is experiential and/or formative asymmetry between the accompanying and the accompanied, the process contributes to the personal development of both (51), if not even more so for the one accompanying (43).

#### 3.2.5 Accompaniment humanizes relationships

Accompaniment is an anthropological response that humanizes relationships (44), as it shows “our way of being human and our way of life” enabling the integral development of people (49), recalling “their true nature, their transcendent and eternal dimension” and facilitating healing (42).

It responds to the human relational condition considered from a personalist model (44, 48) which calls one to accompany and also be accompanied (49). It helps discover the goodness that can be found in human relationships (49). It takes human beings back to their ontological interdependence, generating connection and a mode of existence in accordance with their own inherent structure or nature (50). From this perspective, dependency can be seen as an opportunity (49). In its concrete expression, accompaniment is a positive symbol in the face of the severing of ties (21).

Accompaniment humanizes relationships because it does not necessarily focus on one area (family, school, work, therapy…) and does not depend on any certain age (51). It is not incompatible with certain technically defined practices - in fact, it can be situated as a way of relating and being that may be incorporated into the heart of these practices (21). In this sense, it can be understood as a methodological aid that can enrich such practices (39). It is a form of relationship that cuts across professions, disciplines and different contexts of people’s lives (43).

#### 3.2.6 Synthesis of defining attributes

Based on the frequency and conceptual centrality of the attributes identified in the literature (Table 1), accompaniment can be synthesized as a personal, non-hierarchical and ethically grounded relationship, oriented towards the integral development of the person, unfolding through shared path or process over time, that generating a humanizing relational climate.

These five attributes constitute the minimal and necessary core of the concept, allowing it to be clearly differentiated from related professional relationships such as mentoring, tutoring, counseling, coaching or supervision. Their joint presence is required for a relationship to be considered accompaniment in a strict conceptual sense; the absence of any of them would imply a different type of relational practice (Table 2).

**TABLE 2:** Synthesis of the defining attributes.

Accompaniment attributes	Synthesis
It is a personal relationship	It is an egalitarian relationship focused on someone (not on something), being with and for another in an intentional and non-directive way
It is oriented toward personal development or growth	It responds to the human condition of being open to the flourishing of all human faculties and dimensions, addressing the individual as a whole.
It is an ethical relationship	The person who accompanies takes on a moral responsibility, experiences genuine respect for the dignity of the other, and takes care for the other by being present in a compassionate way.
It is path or process	It is a shared journey that unfolds over time, enabling mutual growth and encounters with others, oneself, reality and the transcendent, all the while remaining open to the unpredictable.
It humanizes relationships	It is an anthropological response that can transcend professions, disciplines, and contexts, helping us to understand what it means to be human revealing the value of relationships, where dependency can be an opportunity.

### 3.3 Proposed definition

According to the results of this conceptual analysis, the definition of accompaniment is as follows:

*Accompaniment is a form of relationship between two or more persons who share a path or process over a significant period of time, oriented towards reciprocal development, in which at least one of them intentionally offers presence, acceptance, appreciation and support in order to foster the full and integral growth of the other, while recognizing and respecting their dignity, freedom and personal context.*

### 3.4 Model cases in the healthcare sector

In the area of healthcare, a model case is that of accompaniment in palliative care, which includes all the attributes of the concept: a personal, ethical relationship, oriented toward the development of the person through a process that fosters and nurtures the humanization of relationships. In this case, the possibility of growth is especially relevant, with a challenge to accompany the patient in a process of recognizing an experience of meaning for one’s own life, the interpersonal relationship and the relationship with the transcendent or sacred (42). The song “*Eso que tú me das*” (*What you give to me*) by the band Jarabe de Palo is a paradigm of the meaning of accompaniment from the patient’s perspective (57).

In the area of health education, a model case is the educational trip to the *Traveling Seminar on Nazi Medicine* (58). This intervention, which, like the palliative case, includes all the attributes of the concept, is an active and experiential teaching experience aimed at the students’ personal development. It consists of a 5-day trip visiting Nazi medical memorials as well as group activities reflecting on the experience. Students are accompanied by a team of teachers who provide the opportunity for holistic personal development by acquiring new perspectives, opinions and a critical sense from a historical, political, health, ethical and relational point of view.

In the area of management, at the Congress *Accompaniment in the Healthcare World* (59) the person responsible for the medical management of a hospital shared his approach to management, which included all the attributes of the concept of accompaniment. For him, management accompaniment is achieved by establishing a personal relationship. Not only that: when he takes on the supervisors under his responsibility, he endeavors to make it easier for them to become “the best people possible.” This generates a dynamism in which both the manager and his team embark on a process of personal growth. Considering that the hospital is “the place on the planet where the most unforgettable emotions occur per square meter and per second,” accompanying in management means nourishing daily work with a purpose or a mission. Its fundamental element is care, which “is what makes us truly human.” This attitude makes people grow, as it makes them pay attention to small details in order to improve.

### 3.5 Additional cases

#### 3.5.1 Border line cases

##### 3.5.1.1 Vocational counseling

Borderline cases are those where there is overlap in many aspects of the concept’s attributes. However, some small difference is to be found. In the article entitled *An Operational Definition or Rehabilitation Counseling* (60) the author provides a definition of the concept *vocational counseling*. A review of the attributes of this concept shows that they coincide point by point with almost all the attributes of the “*accompaniment”* concept. However, there is a relevant difference: Lofquist points out that the “*vocational counselor performs in the role of the expert on techniques for discovering data relevant to the vocational planning process”* (60). It is important to note that this aspect is not included in the core elements of the accompanying person’s role. Accompaniment, as it has been proposed in this work, would be within the reach of people without specific training, even though this training can greatly enrich the practice of accompaniment.

##### 3.5.1.2 Mentoring

In mentoring, the mentor promotes a situation that facilitates the personal and professional development of the mentee, seeking ways to succeed by focusing on development in areas such as career progression, academic achievement and personal development (61).

Mentoring and accompaniment are related cases because they share some attributes such as the pursuit of personal development, being an ethical relationship in which a good is offered and the fact that they are carried out within a process. Nevertheless, it differs fundamentally in that a necessary condition for mentoring - but not for accompaniment - is that it is oriented towards the professional development of the mentoree: mentoring takes place throughout a teaching-learning process in which competencies, professional identity and professional role development are generated with the mentor as an example to be followed (62). However, when *mentoring* is open to a holistic development of the person, and not only professional, it incorporates the notion of accompaniment as it has been understood and taken up in this work. It is a paradigmatic situation in which it can be seen that the notion of accompaniment presented here can be transversal to various practices.

#### 3.5.2 Related cases

##### 3.5.2.1 Coaching

Historically, the classic case of coaching is the act of preparing an athlete for competition (63). Its application in psychology generally consists of a collaborative, individualized, solution-focused, results-oriented process that encourages self-directed learning and is based on the scientific evidence of psychology, while incorporating ethical professional practices (64).

Coaching and accompaniment are related concepts as they share the fact that it is a personal relationship in which a certain good is ethically offered. However, they differ in that in coaching there is necessarily a specific objective to be achieved, a solution that must be accompanied by results and that requires specific professional training - be it sports coaching (63), psychological care (64)., occupational nursing (65) or health-related assistance (66). Accompaniment, as we have seen, is oriented towards the integral development of the person.

##### 3.5.2.2 Counseling

*Counseling* shares several attributes with the concept of accompaniment, with key elements such as active listening, empathy, respect, acceptance, in the context of a person-centered helping relationship (67).

However, it focuses on the field of psychology with a repertoire of psychological interventions that require specific training. Moreover, the objective is not necessarily the growth of the whole person. Rather, it focuses on modifying psychic processes aimed at healing, problem management, managing transitions and crises and developing life skills (67). Counseling has been incorporated into activities such as pastoral counseling (68).

##### 3.5.2.3 Motivational interview

The main difference between “motivational interviewing” and “accompaniment” is that the former is based on a communication skill oriented towards a communicational style, rather than a person-to-person relationship, as in the case of accompaniment. It is oriented “*to strengthen personal motivation for and commitment to a specific goal by eliciting and exploring the person’s own reasons for change*” (69). This is not an aspect to accompaniment, which is open to change or non-change, to a personal type of relationship; this is a goal-oriented, problem-solving approach in which motivation plays an important role.

##### 3.5.2.4 Preceptorship

It is “*an individual teaching/learning method in which each student is assigned to a particular preceptor… so he/she can experience day-to-day practice with a role model and resource person immediately within the clinical setting”* (70). In this sense, it focuses on the educational sphere with a goal that “each student achieves the highest level of educational proficiency and competence possible” (71). It requires a formalization of roles (72), which is not strictly necessary in accompaniment. In this case, the mentoring is characterized by a very specific style of relationship, orientation and process, which is different, although it can be enriched by the attributes of accompaniment.

##### 3.5.2.5 Shared decision making

“*Shared decision making occurs when two autonomous and uncoerced agents both commit to actions that neither has reason to want to change based on their understanding of anticipated outcomes given the situation at hand and of the intended actions of the other party”* (73). In this process, there does not necessarily have to be a strictly personal relationship, nor does it have to be oriented toward personal vocational growth as in accompaniment, but a pragmatic relationship, oriented toward the resolution of a certain problem through a deliberate process. All of this can be enriched by accompaniment or enrich a given accompaniment process.

##### 3.5.2.6 Clinical supervision

Clinical supervision takes place specifically in a working relationship context, which does not necessarily occur with accompaniment, and is aimed at managing, supporting, developing and evaluating the work of colleagues. The objectives are “normative” (e.g., quality control), “restorative” (e.g., encouraging emotional processing) and “formative” (74).

#### 3.5.3 Contrary cases

##### 3.5.3.1 Unwanted loneliness

Loneliness has been defined as a “debilitating psychological condition characterized by a profound sense of emptiness, worthlessness, lack of control and personal threat” (75). It is associated with negative health consequences (76). Unwanted loneliness does not meet any of the attributes of the concept of accompaniment.

##### 3.5.3.2 Self-sufficient dominant individualism

This is manifested when someone who in their relationships exclusively seeks their own self-interest and self-sufficient independence, seeing others only as an obstacle or as a means to achieving their goals, establishing processes based on domination, without regard for possible harm or other negative side effects for others, unless it is to their own detriment. This case also clearly does not meet any of the attributes of accompaniment.

### 3.6 Antecedents to accompaniment

Antecedents are the events (occurrences) or incidents (unpleasant or unusual events) that must occur before the concept is produced.

The main antecedent is the human condition itself. Human beings are open to and in need of others from the very first moments of their existence (77). Hence, it can be said that care is a relational activity that is necessary for humans to develop their possibilities (78). Accompaniment is a form of care that attends to the complexity of the human biological, psychological, relational and spiritual constitution (79).

The human condition translates into the need for support in order to obtain basic needs and survive, but also the need to take advantage of an individual’s best possibilities, happiness being the possibility of all possibilities - that is, the possibility of opening up to the perfection of human reality itself (80). People need to find purpose and meaning in their existence: to understand that their life has a direction and that it is accompanied by an experience of fulfillment, that life is worth living, despite difficulties and setbacks. Moreover, people ask to be treated as such and not as objects or as mere problems to be solved (81).

For accompaniment to take place, at least one person needs to have understood the meaning of the concept, to have considered it to be of value and therefore to have glimpsed what is positive and true in it. It is positive in that it contributes to discovering and achieving the possibilities that best promote human perfection and happiness; true in that it truly contributes to the discovery of the best in each person when it permeates human relationships.

### 3.7 Consequences

Consequences are the events or incidents that occur as a result of the occurrence of the concept of accompaniment.

When someone deliberately establishes a person-to-person relationship, it happens that the person accompanied feels valued and important to the other. This is relevant in any form of relationship, but especially when there is an asymmetry where the partner has a health problem, is the subject of medical research, requires training or is in a lower position in the organizational chart. The “I-Thou” connection, i.e., person-to-person, arises from dialogue, especially dialogue related to life experiences. These experiences may seem trivial and useless from a pragmatic point of view oriented to the resolution of health problems; however, they are the vital channel necessary for the establishment of a personal encounter (82).

The fact that the relationship is ethical implies that the companion assumes a moral responsibility for the relationship. The consequences will therefore be that accompaniment should not result in any intentional harm (physical, psychological, relational or spiritual) to the person being accompanied, but in good - at least in the form of wellbeing and, at its best, in the form of a process of personal growth. In the context of any discipline or profession, accompaniment should not violate the values and principles of that practice. In a work context, accompaniment modifies the way of relating: it turns from a merely functional and pragmatic type of relationship to a type of personal relationship in which the person being accompanied sees his or her desire or vocation for personal and professional development recognized and channeled, whenever possible.

Relationships, thanks to a culture or climate of accompaniment, will gain in wellbeing, harmony, collaboration, meaning and mutual commitment.

### 3.8 Empirical referents

Empirical referents are real phenomena that reflect the occurrence of the concept of accompaniment. The defining attributes may coincide with referents. Certain behaviors would be empirical referents, as well as the expression of experiences associated with the attributes of the concept. Empirical references are useful for a later stage of research that is beyond the scope of this study. They can be used to design items for a measurement scale that assesses how the concept appears in real-life situations.

As a relationship, time and space are shared, as well as communication between at least two people. As it is a personal relationship, the companion is able to recognize that they see the other as a person and not as a problem to be solved. The person being accompanied, meanwhile, may reflect that they have felt treated as a person and not as an object or a mere problem. In communication, the accompanied person recognizes that they can express themselves freely enough and that he/she feels treated as an equal, not merely as a role bearer (e.g., subordinate, pupil or patient) within the social context in which he/she finds him/herself.

As it is an ethical relationship, the companion can recognize that they feel and act with respect for the dignity of the person being accompanied and that they feel a genuine interest in the other and their experiences. Moreover, they may recognize that they deliberately seek the good of the other by trying to care for them in every moment of interaction. An attitude of welcoming the fragility of others, whatever its manifestation, is also experienced. The accompanied person is able to recognize a treatment marked by respect and consideration, and does not feel judged, belittled or disqualified and recognizes the availability of their companion to find moments of encounter and that the relationship between them is a good thing. The person being accompanied appreciates that their values, preferences and times needed in the taking of decisions are respected. Moreover, they do not feel manipulated or pressured when doing so.

By being oriented toward a path of personal development, the person being accompanied can recognize that, in the context in which they move, their best aspirations are recognized, valued and ways are sought for them to be reached. They may also be able to appreciate moments when their situation or way of acting is questioned in order to look for areas of improvement. The accompanied person feels that personal problematic situations are taken into account and welcomed, even if they are not necessarily directly related to the context. He or she may acknowledge that their ideas, convictions, feelings, desires or needs are taken into account.

As it is a path or process, the person being accompanied can recognize a temporal continuity in the relationship and that in this continuity, changes are taking place that contribute to their development. Also, they will reflect that the relationship takes place in a climate of trust, of personal encounters and, occasionally, of disagreements in which their beliefs and understandings are questioned with sound reasoning, in order to discover possibilities for improvement. They recognize that it is a process in which they can share experiences with their companion. In this process, the companion recognizes that they are also going through a process of personal growth.

As a process that humanizes relationships, in a culture of accompaniment, both the companion and the accompanied can recognize that relationships tend to move in a climate of kindness, respect and trust. They recognize that they discover goodness and a sense of existential fulfillment through a relationship of accompaniment (as opposed to other forms of relationship), and they can see that this type of relationship offers diverse opportunities. They also appreciate that the relationship of accompaniment enriches technically defined practices.

## 4 Discussion

In this systematic review, we identified academic publications addressing the concept of accompaniment, which began to appear more frequently in the first decade of the 21st century. The most recurrent defining attributes describe accompaniment as a personal and ethical relationship oriented towards human development, unfolding through a shared process in which a relational climate is generated that fosters the humanization of interactions.

Model and additional cases were drawn from palliative care, healthcare training and team management, while contrary cases included experiences of unwanted loneliness and self-interested, individualistic behavior. Related constructs—such as mentoring, coaching, counseling, motivational interviewing, preceptorship and shared decision-making—were also examined, allowing their similarities and differences with accompaniment to be clarified.

The openness and vulnerability inherent in the human condition emerged as a key antecedent of accompaniment. Regarding consequences, accompaniment is associated with the experience of being valued, with deliberate efforts to avoid harm and promote the good of the other, and with the potential to cultivate relational cultures grounded in wellbeing, collaboration, harmony and mutual commitment.

Observable manifestations of accompaniment include the intentional sharing of time, space and process, in which the accompanied person perceives respectful treatment, personal recognition and support for their life aspirations, within a climate characterized by trust, closeness and human warmth that enriches technical and professional practice.

This understanding of accompaniment may be compared with Lofquist’s early work (1959), who, under the framework of “rehabilitation counseling,” described a continuous, non-authoritarian learning process oriented toward vocational planning. Although framed in a different disciplinary context, this definition anticipates several attributes later associated with accompaniment, particularly its relational, developmental and non-directive character. The notion of vocation, understood as the expression of human aspirations and a path toward personal fulfillment, further connects accompaniment with the anthropological condition of openness to growth.

The first systematic conceptual study of accompaniment was conducted by Maela (21), who documented its expansion across professional fields since the 1990s and defined it as a “specific professional position”. For Maela, accompaniment constitutes an institutionalized, reflexive and ethically grounded relationship, characterized by the intentional presence of the companion, the refusal to impose goals and the respect for the autonomy and decision-making capacity of the accompanied person.

While our findings coincide with Maela in recognizing accompaniment as a specific relational phenomenon distinct from tutoring, mentoring or counseling, our analysis extends the concept beyond strictly professional contexts, showing that accompaniment may also occur in non-professional settings, provided that it is grounded in appropriate relational competence and ethical commitment.

Simard’s work (2016), focused on social exclusion, further supports this view. He conceptualizes accompaniment not as a profession but as a transversal practice centered on the person rather than on problem-solving, drawing on the relational Philosophies of Lévinas and Buber (82–85). Simard emphasizes the primacy of the interpersonal encounter, equality between participants and the refusal to impose direction, thereby reinforcing the personal and ethical dimensions identified in our analysis.

Similarly, the study coordinated by González Iglesias (49) highlights several conditions for authentic accompaniment, including deep personal understanding, active listening and unconditional acceptance. Additional elements such as forgiveness and hope are proposed as features of a broader community culture of accompaniment. Although these latter aspects were not retained as defining attributes due to their limited recurrence in the literature, they nonetheless enrich the conceptual landscape and point to relevant areas for future research.

At the Universidad Francisco de Vitoria, where this study was conducted, accompaniment constitutes a foundational element of the institutional educational model, permeating mentoring programs across faculties. This context illustrates how mentoring, traditionally oriented toward professional development, can be enriched by an accompaniment approach that emphasizes personal growth, relational depth and ethical presence.

From an anthropological perspective, accompaniment involves the intentional offering of presence, recognition and support, grounded in respect for human dignity and freedom. It presupposes a voluntary disposition to walk alongside another, fostering trust, unconditional acceptance and the conditions for integral development. Such relationships generate a safe and meaningful relational climate that contributes to the humanization of professional practices and to the wellbeing of both individuals and communities.

In synthesis, accompaniment emerges as a person-to-person process oriented towards integral development and vocational discernment, unfolding through a shared path that humanizes relationships and responds to the relational nature of the human being. While it requires specific competencies and ethical formation, fostering a culture of accompaniment may represent a key strategy for enhancing the humanization of healthcare in clinical, educational, research and managerial contexts.

This study has several limitations. Despite an exhaustive search, relevant publications may have been missed. The inclusion criterion requiring an explicit original definition of accompaniment may have led to the exclusion of sources describing important attributes without formal conceptualization. Moreover, given the richness and multidimensionality of the concept, some relevant aspects emphasized in monographs or theoretical works may not have been captured. As with any conceptual analysis, the present findings should therefore be considered provisional and open to further refinement and empirical validation over time.

## Data Availability

The original contributions presented in this study are included in this article/Supplementary material, further inquiries can be directed to the corresponding author.

## Footnotes

1
https://www.rayyan.ai/
