# Supplementary material for: Accompaniment in the healthcare sector: a systematic review and concept analysis
Source: Front Med (Lausanne). 2026 Feb 20;13:1724133. doi: 10.3389/fmed.2026.1724133 (PMC12962899; doi:10.3389/fmed.2026.1724133)
Supplement: Supplementary file 1 [file Supplementary_file_1.docx]

Pubmed

"Accompaniment"[Title] OR "mentors"[MeSH Terms] OR "mentoring"[MeSH Terms] OR "mentoring"[MeSH Terms] OR "counseling"[MeSH Terms] OR "preceptorship"[MeSH Terms] OR "decision making, shared"[MeSH Terms]) AND ("definition"[Title] OR "construct"[Title] OR "scale"[Title])

Embase:

('accompaniment':ab OR coaching:ab OR mentoring:ab OR counseling:ab OR 'shared decision making':ab OR preceptorship:ab ) AND (definition:ab OR construct:ab ab OR concept:ab OR 'validated scale':ab)
