# Supplementary material for: Accompaniment in the healthcare sector: a systematic review and concept analysis
Source: Front Med (Lausanne). 2026 Feb 20;13:1724133. doi: 10.3389/fmed.2026.1724133 (PMC12962899; doi:10.3389/fmed.2026.1724133)
Supplement: Supplementary file 2 [file Supplementary_file_2.docx]

| **Author** | **Study type** | **Contexto** | **JBI Quality Assesment** | | | | | |  |  |  |
| --- | --- | --- | --- | --- | --- | --- | --- | --- | --- | --- | --- |
|  |  |  | **Item 1** | **Item 2** | **Item 3** | **Item 4** | **Item 5** | **Item 6** |  |  |  |
| **Abanto Zorrila, 2012** | Textual evidence: expert opinion | Strengthen teaching performance and create harmonious learning environments to benefit both teachers and students | Y | U | Y | Y | Y | Y |  |  |  |
| **Aubert, 2009** | Textual evidence: expert opinion | To support adults in the process of validating their experience, focusing on their needs and benefits | Y | Y | Y | Y | Y | Y |  |  |  |
| **Barahona, Medina, & Sastre, 2019** | Textual evidence: expert opinion | It approaches accompaniment from a philosophical and relational perspective, involving and benefiting the community as well as educational and social structures | Y | Y | Y | Y | Y | Y |  |  |  |
| **Barbero et al., 2016** | Textual evidence: expert opinion | To improve the comprehensive care of patients with advanced diseases, their families, and the professional care team | Y | Y | Y | Y | Y | Y |  |  |  |
| **Barbot, 2004** | Textual evidence: expert opinion | It analyzes the trainer–learner relationship in educational and digital contexts, focusing on learner empowerment | Y | Y | Y | Y | Y | Y |  |  |  |
| **Batiz, 2023** | Textual evidence: expert opinion | Addressed to healthcare professionals and family members, with the aim of incorporating emotional, interpersonal, and compassionate factors into end-of-life care | Y | Y | Y | Y | Y | Y |  |  |  |
| **Castaño Muñoz, García Cardo, & Díaz López, 2023** | Textual evidence: expert opinion | To support those who provide accompaniment in the university, promoting relational and educational improvement among teachers and mentors | Y | Y | Y | Y | Y | U |  |  |  |
| **Farmer, 2012** | Textual evidence: expert opinion | To work with and through public institutions for the benefit of the local population | Y | Y | Y | Y | U | Y |  |  |  |
| **González Iglesias, 2023** | Textual evidence: expert opinion | To train and support individuals in different areas (educational, professional, and personal | Y | Y | Y | Y | Y | Y |  |  |  |
| **Guité-Verret et al., 2023** | Textual evidence: expert opinion | To improve the quality of accompaniment for patients at the end of life, benefiting both clinicians and patients | Y | Y | Y | Y | Y | Y |  |  |  |
| **Maela, 2004** | Textual evidence: expert opinion | It offers reflections for trainers, therapists, educators, and counselors who practice accompaniment in the fields of education, health, and the social sector | Y | Y | Y | Y | Y | Y |  |  |  |
| **Orón Semper & Cenoz Larrea, 2023** | Textual evidence: expert opinion | To use personal accompaniment through dialogue to foster the growth of students, teachers, and mentor | Y | Y | Y | Y | Y | Y |  |  |  |
| **Pope, 2019** | Textual evidence: expert opinion | It advocates an ethics of accompaniment aimed at the service and empowerment of marginalized communities | Y | Y | Y | Y | Y | Y |  |  |  |
| **Puchalski, 2020** | Textual evidence: expert opinion | It focuses on compassionate presence with patients and families, emphasizing their dignity in the midst of suffering | Y | Y | Y | Y | Y | Y |  |  |  |
| **South African Nursing Council, 1992, como se cita en Rikhotso et al., 2010** | Textual evidence: expert opinion | To establish policies and a philosophy aimed at safeguarding nursing education, benefiting students, patients, and the healthcare system alike | Y | Y | Y | Y | Y | Y |  |  |  |
| **Simard, 2016** | Textual evidence: expert opinion | To support individuals experiencing social disruption, through a human and relational approach that is essential for this population | Y | Y | Y | Y | Y | Y |  |  |  |
| JBI Joana Briggs Institute Check list for textual evidence: expert opinion.Pearson A, Jordan Z, McArthur A, Florescu S, Cooper A, Yan H, Klugarova J, Stannard D, Edwards D. Systematic reviews of textual evidence: narrative, expert opinion or policy (2024). Aromataris E, Lockwood C, Porritt K, Pilla B, Jordan Z, editors. JBI Manual for Evidence Synthesis. JBI; 2024. Available from: https://synthesismanual.jbi.global. https://doi.org/10.46658/JBIMES-24-04 | | | | | | | | | |  |  |
|  |  |  |  |  |  |  |  |  |  |  | |
|  |  |  |  |  |  |  |  |  |  |  | |
| Y(green): yes; N (red): No U (yellow): unclear | | | | | | | | | |  | |
| Item 1: Is the resource of the opinion clearly identified? Are the authors clearly identified ( including their name, roe, experience and qualifications? | | | | | | | | | |  | |
| Item 2: Does the source of the opinion have stannding in the field of expertise? Determinning whether the aouthos is informed or possesses knowledge about the specific subject. | | |  |  |  |  |  |  |  | |  |
| Item 3: Are the interest of the relevant population the central focus of the opinion? The expert opinion should focus on improving outcomes, and it is important to determite that the opinion has such a focus. | | | | | | | | | |  | |
| Item 4: Does the opnion demonstrate a logically defended argument to support the conclusions drawn | | | | | | | | | |  | |
| Item 5: Is the refrence to the extant literature? | | | | | | | | | |  | |
| Item 6: Is there any incongruence with the literauture / sources logically defended? Has the author demonstrate awareness of alternate or dominant opinions in the literature? | | | | | | | | | |  | |
